# Retraction Note: MiR-362-5p promotes the malignancy of chronic myelocytic leukaemia via downregulation of GADD45α

**DOI:** 10.1186/s12943-022-01573-1

**Published:** 2022-04-04

**Authors:** Peng Yang, Fang Ni, Rui-qing Deng, Guo Qiang, Hua Zhao, Ming-zhen Yang, Xin-yi Wang, You-zhi Xu, Li Chen, Dan-lei Chen, Zhi-jun Chen, Li-xin Kan, Si-Ying Wang

**Affiliations:** 1 Department of Pathophysiology, School of Basic Medical Science, Anhui Medical University, 81 MeiShan Road, Hefei, Anhui 230032 PR China; 2 Department of Transfusion, the First Affiliated Hospital of Anhui Medical University, Hefei, Anhui 230022 PR China; 3 Department of Haematology, the First Affiliated Hospital of Anhui Medical University, Hefei, Anhui 230022 PR China; 4 Department of Clinical Medicine, Anhui Medical University, Hefei, Anhui 230032 PR China

**Retraction Note: Mol Cancer 14, 190 (2015)**

**https://doi.org/10.1186/s12943-015-0465-3**

The Editor in Chief retracted this article because of significant concerns regarding a number of Figures presented in this work, which question the integrity of the data. The Editor-in-Chief therefore no longer has confidence in the integrity of the data in this article.

Peng Yang, Ming-zhen Yang, Danlei Chen & Si Ying Wang have agreed to this retraction but not to the wording of this retraction notice. Fang Ni, Rui-qing Deng, Guo Qiang, Hua Zhao, Xin-yi Wang, You-zhi Xu, Li Chen, Zhi-jun Chen & Li-xin Kan have not responded to any correspondence from the editor/publisher about this retraction.

